# Spatial and Temporal Evolution and Driving Factors of Urban Ecological Well-Being Performance in China

**DOI:** 10.3390/ijerph19169996

**Published:** 2022-08-13

**Authors:** Jing Bian, Feng Lan, Yulin Zhou, Zhenzhen Peng, Mingfang Dong

**Affiliations:** 1 School of Management, Xi’an University of Architecture and Technology, Xi’ an 710055, China; 2 Research Center of Green Development and Mechanism Innovation of Real Estate Industry in Shaanxi Province, Xi’an University of Architecture and Technology, Xi’an 710055, China

**Keywords:** ecological well-being performance, super-SBM model, spatial Durbin model, sustainable development

## Abstract

Extensive development leads to the decline of ecological well-being, and it is necessary to improve the urban ecological well-being performance (EWP). This paper adopted the Super-slack-based measure (Super-SBM) model to evaluate the EWP of 285 Chinese prefecture level cities from 2011 to 2017. The exploratory spatial data analysis method (ESDA) was used to explore the spatial and temporal evolution characteristics of the EWP, and then the spatial Durbin model (SDM) was adopted to analyze the driving factors of the EWP. The results show that the trend of the overall average EWP has experienced a stage evolution process of “upward → downward → upward”. The urban EWPs have significant spatial agglomeration and path dependence. The economic development level and technological progress had the positive impacts on the EWP, and the urbanization level, economic extroversion and industrial structure had the negative impacts on the EWP. The result reveals that there was a “U-shaped” relationship existing between urbanization level and the EWP. The negative spatial spillover effect of urbanization level on the EWP was significant. The corresponding policy implications were put forward. This study will provide strategic guidance for policy makers to optimize and enhance the urban EWP.

## 1. Introduction

**Figure 1:**
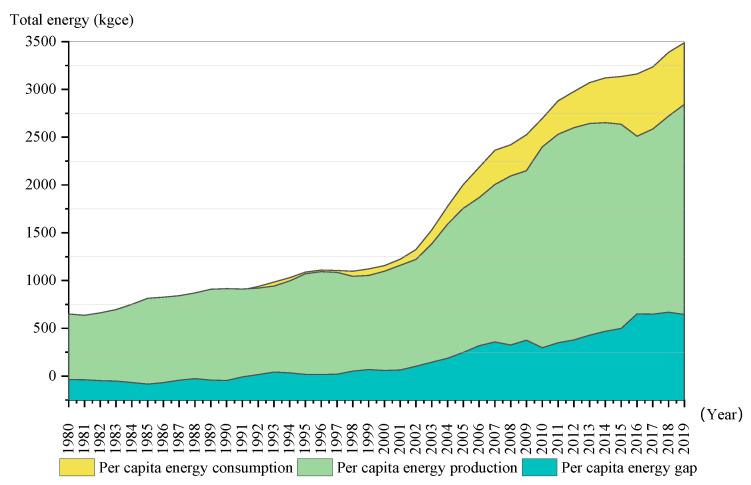
China’s per capita energy gap from 1980 to 2019.

The rapid urbanization development in China has made significant progress, but it has also faced many challenges. Environmental deterioration, a lack of resources, a shortage of resources and other ecological problems have seriously damaged the ecosystem, which has hindered the promotion of people’s well-being and the ecological civilization. The proportion of population who are urban residents will raise to 68% in 2050 [1]. With the rapid increase of global population, the problem of resource consumption has become increasingly serious [2]. As shown in Figure 1, the total energy consumption per capita in China was 3488 kgce in 2019, nearly 5.68 times those of the total consumption in 1980 [3]. Meanwhile, the problems of “urban diseases” appear, for example, as a result of insufficient water supply, urban traffic congestion, low land use efficiency, insufficient public infrastructure construction and so on, which bring severe challenges to urban development. Climate change, ecological environmental damage and other issues are intertwined, affecting people’s health, economic and other well-being levels in different ways [4,5].

China has actively been promoting a new type of urbanization for solving serious ecological environment problems and improve people’s well-being in recent years. China’s urban construction has changed from extensive development to a new type of urbanization. The 14th Five-Year Plan (2021–2025) clearly proposed the goal of achieving new progress in the building of a Beautiful China and a new level of people’s livelihood. The economic system is subordinate to the ecological system, rather than an independent and unrestricted system [6]. Ecological well-being is the expansion of the connotation of social well-being, which has raised sustainable development and quality of life to a more important position [7]. Ecological well-being performance (EWP) refers to the efficiency of transforming natural consumption into human well-being [8]. Therefore, it is an inevitable tendency for promoting the EWP and high-quality development under the constraints of ecological resources.

The research on the EWP is developing in the continuous global search for a healthy and benign economic development model, and has highly concerned scholars in recent years [9]. Some of the existing literature has carried out comprehensive analysis of the EWP evaluation, space distribution of the EWP and its driving factors. The evaluation methods of the EWP mainly include two types [10]. First is the single-factor evaluation method, and the proportion of well-being level to natural consumption is used to estimate the EWP [11]. Well-being level is usually measured by the Human Development Index (HDI) or average life expectancy, and ecological consumption is usually measured by ecological footprint (EF) [12]. For example, Daly initially used the ratio of natural consumption (service) to well-being level (ecological resource throughput) for calculating the sustainable development level of a national region [13,14]. Abdallah et al. [15] put forward the Happy Planet Index (HPI), which measured the longevity and happiness of life. The other is comprehensive evaluation method, such as Data Envelopment Analysis (DEA) and Stochastic Frontier Analysis (SFA) [16]. For example, Hu et al. used the network DEA model to estimate the EWP, and analyzed the evolution and impact of the urban EWP in the Yangtze River Delta [17]. Xiao et al. adopted the improved SFA model to estimate the EWP of 79 Chinese cities in the Yellow River Basin [18]. Bianchi et al. used the DEA model to assess the comparable development of eco-efficiency and the geographical heterogeneity in the European region between 2006 and 2014 [19]. Moutinho et al. used a two-stage DEA method to measure the eco-efficiency scores of European Union countries from 2008 to 2018 [20].

There are great differences in economic development, resource endowments and energy consumption in different Chinese regions, and different regions have spatial differences in terms of the EWP. For example, Wang et al. explored the spatial and temporal differentiation characteristics of the EWP of 30 provinces in China [21]. Li et al. selected the Yellow River Delta as the research area, and adopted the coupling coordination degree model to research spatiotemporal evolution, and used the geographic weighted regression model to analyze the influencing mechanism of the EWP [22]. Deng et al. used the kernel density estimation method and Markov chain to reveal the spatial disequilibrium and dynamic evolution characteristics of the EWP [23]. Zheng et al. used the exploratory spatial data analysis (ESDA) method to explore the spatial-temporal distribution pattern of the EWP in China [24]. Kounetas et al. used a nonparametric model to estimate patterns of convergence or divergence of environmental performance in U.S. states from 1990 to 2017 [25]. It has become an important direction to carry out the spatial and temporal evolution research of the EWP and simulate the change trend of the EWP.

Many studies have been conducted to explore the driving factors of the EWP. The traditional quantitative methods such as Tobit model, Logarithmic Mean Divisia Index (LMDI) method and other econometric model have been extensively employed to explore driving factors of the EWP. For example, Dietz et al. [26] studied the relationship between human well-being environment intensity and economic growth in 58 countries, and they argued that the result was opposite to Kuznets curve. Jorgenson, et al. [27] analyzed the dynamic relationship between human well-being energy intensity and economic growth in 12 European countries. Behjat and Tarazkar [11] employed the autoregressive distributed lag model to reveal influencing factors of the EWP in Iran from 1994 to 2014, finding that the population growth has a negative effect on the EWP. Silva et al. selected Brazil as a case study, and adopted spatial regression models to explore the impacts of the affluence and income inequality on the environmental intensity of well-being [28]. Ahmed [29] analyzed the driving factors of natural resources abundance, human capital, and urbanization on the ecological footprint in China. Xiao and Zhang [30] used Tobit model to explore the influence mechanism of the EWP, and the study showed that green technology innovation efficiency had a positive impact on the EWP.

By summarizing the existing literatures, the research of the EWP evaluation, space distribution and driving factors are in the stable increase period, and there are relatively rich research results and considerable research foundation. However, there are still some deficiencies in relevant literatures. The EWP study mainly focuses on the national and provincial scales, but pays little attention to the urban EWP. The data of these scales have some limitations in reflecting the internal heterogeneity of regional development, so it is essential to carry out the research on urban EWP. Furthermore, the spatial and temporal evolution characteristics of the EWP has not been sufficiently explored, and the spatial effect of driving factors on the EWP needs to be further considered. The traditional econometric analysis usually ignores the existence of spatial correlation and does not consider whether the EWP has the effects of spatial spillovers, affecting the accuracy of analysis results. Consequently, it is necessary to study the spatial and temporal evolution characteristics of the EWP and analyze the spatial effects of driving factors on the EWP.

Therefore, the aims of this study were: (1) to use the Super-SBM model for estimating the EWP of 285 prefecture-level cities in China, and to propose the ESDA method for exploring the spatial and temporal evolution characteristics of the EWP; (2) to adopt spatial econometric model for analyzing driving factors of the EWP; (3) to design strategies for enhancing the EWP with urban characteristics. The major novelty of this study is summed up as follows. (1) Existing literatures usually ignores the spatial effects of the urban EWP. The ESDA method is employed in this study, which can reveal spatial agglomeration characteristics of the EWP. (2) On the basis of considering temporal heterogeneity and spatial heterogeneity, the spatial panel Durbin model is used to test the spatial spillover effect of the EWP, which further enriches the theoretical research in this field. (3) The EWP presents a new study perspective for urban sustainable construction, and the analyzed results and policy implications are conducive to accelerate the implementation of urban management decisions and promote regional sustainable development. 

## 2. Method and Data

### 2.1. The Super-SBM Model with Undesirable Outputs

The Super-SBM model is used to evaluate the EWP, which can also break through the shortcomings of traditional DEA. It consists of the following aspects [31,32]:

(1) Assuming that there are *n* cities (decision-making unit, DMUs) with the X input and Y output matrices. It can be expressed as below:

$$X=(x_{ij})\in R^{m\times n}>0,\;Y=(y_{ij})\in R^{s\times n}>0$$

Then, the EWP production possibility set (P) is expressed as:

P={(x,y)|x≥Xλ,y≤Yλ,λ≥0}

where λ is the non-negative vector in $R^{n}$.

The DUM $\left(x_{0},y_{0}\right)$ can be described as

$$x_{0}=X\lambda +s^{-}$$

$$y_{0}=Y\lambda -s^{+}$$

With λ≥0, $s^{-}\geq 0$ and $s^{+}\geq 0$. The vectors $s^{-}\in R^{m}$ and $s^{+}\in R^{s}$ are the input redundancy and insufficient output. Applying $s^{-}$ and $s^{+}$, the EWP ρ is as below:

(1) $$\min\rho =\frac{1-\frac{1}{m}\sum_{i=1}^{m}s_{i}^{-}/x_{io}}{1+\frac{1}{s}\sum_{i=1}^{s}s_{i}^{+}/y_{io}}$$

(2) $$\mathrm{Subject}\;\mathrm{to}\;\{\begin{array}{c} x_{0}=X\lambda +s^{-}\\ y_{0}=Y\lambda -s^{+}\\ \lambda \geq 0,s^{-}\geq 0,s^{+}\geq 0 \end{array}$$

where ρ is the EWP value, 0<ρ≤1; and λ is the linear coefficient of a DMU; x and y are input and output variables respectively; m and s are the number of input and output indicators respectively.

(2) The Super-SBM model is shown as follows:

(3) $$\delta^{*}=\min\delta =\frac{\frac{1}{m}\sum_{i=1}^{m}\bar{x_{i}}/x_{io}}{\frac{1}{s}\sum_{r=1}^{s}\bar{y_{r}}/y_{ro}}$$

(4) $$\mathrm{Subject}\;\mathrm{to}\;\{\begin{array}{c} \bar{x}\geq \sum_{j=1,\neq 0}^{n}\lambda_{j}x_{j},\\ \bar{y}\leq \sum_{j=1,\neq 0}^{n}\lambda_{j}y_{j},\\ \bar{x}\geq x_{o},\bar{y}\leq y_{0}\\ \bar{y}\geq 0,\lambda \geq 0 \end{array}$$

$\delta^{*}$ is the EWP value. The evaluative DMU is relatively effective when $\delta^{*}$ ≥ 1. The higher the $\delta^{*}$ is, the higher the urban EWP is. The evaluative DMU is relatively ineffective when $\delta^{*}<1$.

### 2.2. Spatial Autocorrelation Analysis

There are two important steps to build a spatial econometric model. The first step is to test the spatial correlation, and the second step is to select and establish an appropriate spatial econometric model. It is necessary to check whether the selected variable data has spatial dependence. The exploratory spatial data analysis (ESDA) method was used to verify whether the selected samples have spatial autocorrelation. The global Moran’s I index is given as follows [33,34]:

(5) $$\begin{array}{cc} I=\frac{\sum_{i=1}^{n}\sum_{j=1}^{n}\omega_{ij}(Y_{i}-\bar{Y})(Y_{j}-\bar{Y})}{S^{{}^{2}}\sum_{i=1}^{n}\sum_{j=1}^{n}\omega_{ij}} & \end{array}$$

A Moran scatter plot reflects the correlation of the internal structure of global space and the correlation of local space [35]. The local Moran’ I index is expressed as:

(6) $$I_{i}=\frac{\left(Y_{i}-\bar{Y}\right)}{S^{2}}\sum_{j=1}^{n}\omega_{ij}(Y_{j}-\bar{Y})$$

where $S^{2}=\frac{1}{n}\sum_{i=1}^{n}{\left(Y_{i}-\bar{Y}\right)}^{2}$; $\bar{Y}=\frac{1}{n}\sum_{i=1}^{n}Y_{i}$, n is the number of cities; $Y_{j}$ is the EWP value of city j; $\omega_{ij}$ is the spatial weight matrix.

The rules are as follows: the scope of Moran’s I statistic is [–1,1]. If Moran’s I > 0, it indicates that there is a positive spatial correlation between the research variables, and there is a certain agglomeration phenomenon. It is shown as agglomeration effect of high EWP value among the local city and its adjacent cities (“High-High”), or it is shown as agglomeration effect of low EWP value (“Low-Low”). If Moran’s I < 0, it indicates that it is a spatial negative correlation. The spatial characteristics of the local city and its adjacent cities have spatial disparity, and it shows “High-Low” or “Low-High” spatial agglomeration effect [36,37]. If Moran’s I is zero, it indicates that the research variables are independent and randomly distributed. When |Z|>1.96, P<0.05, the spatial correlation is significant [38].

### 2.3. Spatial Econometric Model

Spatial econometric models mainly include spatial the autoregressive model (SAR), spatial error model (SEM) and spatial Durbin model (SDM). When the spatial lag term of explanatory variables affects the explanatory variables, it is necessary to consider the establishment of SDM [39]. The basic modality of spatial econometric models can be described as follows:

(7) $$y=\lambda Wy+X\beta +WX\theta +\mu_{i}+\delta_{i}+\varepsilon_{it}$$

(8) $$\varepsilon_{it}=\rho W\varepsilon_{it}+\varphi_{it},\varphi \sim N(0,\sigma_{it}^{2}I_{n})$$

where W is the spatial weight matrix, β is the coefficient of the explanatory variable, ρ is the spatial effect coefficient, θ is the impact parameter of the explanatory variable in the adjacent region, $\mu_{i}$, $\delta_{t}$ are the spatial and temporal effects respectively. $\varepsilon_{it}$ is the random disturbance term.

The spatial panel model covers a variety of commonly used models.

(1) θ=0, ρ=0 and λ≠0, it is simplified as SAR model;
(2) θ=0, λ=0 and ρ≠0, it is simplified as SEM model;
(3) λ≠0 and θ=0, it is simplified as SDM model.

Lesage and pace [40] decomposed the impact of explanatory variables in spatial econometric model on explained variables into direct effect, indirect effect and total effect. The direct effect means the effect of explanatory variables on the local cities, and the indirect effect is the effect of explanatory variables on its adjacent cities, namely spatial spillover effect [41,42]. The total effect is the joint effect of the direct effect and indirect effect [43].

### 2.4. The Integrity Research Method

**Figure 2:**
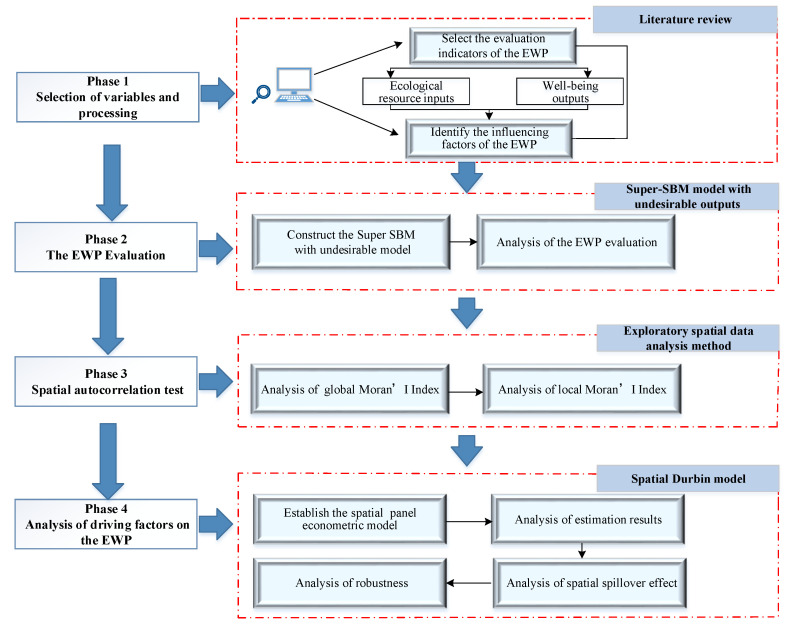
The flowchart of the research methods used in this study.

The integrity research method for this paper is given in Figure 2. First, the index system and driving factors were established through a literature review. Second, the empirical analysis of 285 Chinese prefecture level cities from 2011–2017 was performed, and the Super-SBM model was adopted to evaluate the EWP. Third, the ESDA method was used to explore the spatial temporal evolution characteristics of the EWP. Finally, the SDM was constructed to study the driving factors and spatial spillover effects of the EWP.

### 2.5. Data Collection

According to the data of the National Bureau of statistics, there were data of 298 Chinese prefecture level cities in 2017. Considering availability and accuracy of data, 285 prefecture level cities in China were selected as the research area. Some statistical standards of EWP indicators data changed from 2018 onwards; therefore, the research period of this paper is 2011–2017. The seven years, China has gone through a number of major reform measures, which has a certain theoretical research value. The data are gathered from “China city statistical yearbook 2012–2018” and “China statistical yearbook 2012–2018”. Based on the classification of China’s economic zones by the National Bureau of statistics, 285 cities are divided into the cities of eastern region, middle region and western region.

## 3. Selection of Variables and Processing 

### 3.1. The Selection of the EWP Indicators

**Figure 3:**
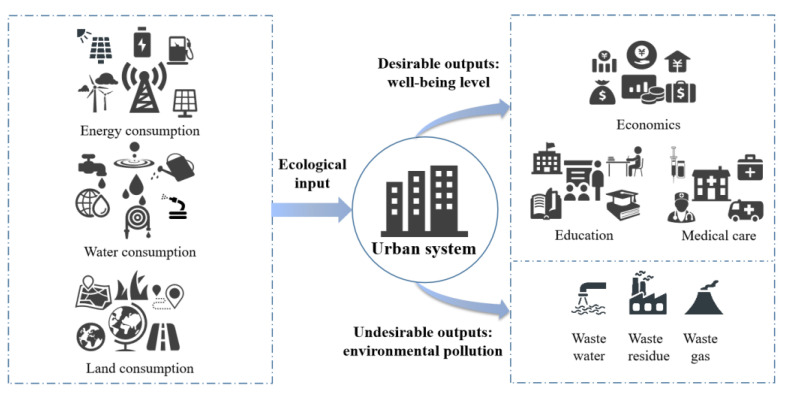
The evaluation indicator structure of the EWP.

This paper constructs the EWP index system structure from the perspective of the two aspects of ecological input and well-being output. Ecological footprint is an appropriate indicator to measure natural consumption. However, it is difficult to obtain data of ecological footprint at urban scale. This paper chooses comprehensive input indicators to measure natural consumption, such as energy, water consumption and land consumption. Objective well-being usually reflects people’s basic economic and environmental needs [44]. The HDI has become widely recognized, which includes education, medical care and economics dimensions. Therefore, referring to that dimension of HDI, this paper proposed the evaluation indicator structure of the EWP, as shown in Figure 3.

#### 3.1.1. The Selection of Input Indicators

Energy consumption. Due to the data of coal, oil and other energy sources at the city level not being available, referring the experience of Zhang et al. [45], the urban electricity consumption ($x_{1}$) is selected to measure energy consumption in this paper.

Water consumption. Water resources are not only the basic element of maintaining human life and health, but also the essential resource in production activities. The water consumption of residential use ($x_{2}$) is adopted to reflect water consumption [46].

Land consumption. Though the spatial expansion of urbanization needs to use a great deal of land, and the total amount of land resources is limited. The built-up area ($x_{3}$) [45] is represented to reflect land consumption in this paper, as shown in Table 1.

#### 3.1.2. The Selection of Output Indicators

(1) Desirable outputs.

The level of economic development. This is often regarded as an important indicator of the economic situation of a country (region); GDP ($y_{1}^{d}$) [42] is selected to reflect urban economic development level in this study.

The level of education development. The number of students enrolled is widely used to measure educational development level. For instance, Shen and Zhou [47] adopted it to characterize the education development level. Consequently, the number of students enrolled per 10^4^ persons ($y_{2}^{d}$) is selected to reflect a city’s educational development level.

The level of medical care. Due to the continuous data on per capita life expectancy in Chinese prefecture level cities is difficult to obtain. The number of doctors per 10^4^ persons ($y_{3}^{d}$) is used to reflect the medical care level [38].

(2) Undesirable outputs.

Considering the availability of data, wastewater, waste gas and solid waste are included in the EWP index system as undesirable outputs. Wastewater discharge ($y_{1}^{\mathrm{ud}}$), SO_2_ ($y_{2}^{\mathrm{ud}}$) and soot/dust emission ($y_{3}^{\mathrm{ud}}$) are selected as undesirable outputs to reflect environmental pollutant indicators [48]. The indicators of this empirical study are the per capita consumption of each sample unit, that is, the total amount of indicators divided by the permanent population.

### 3.2. The Selection of the EWP Driving Factors

#### 3.2.1. Economic Development Level

Economic factors are particularly important for the urban EWP, which reflect the scale or degree of economy development in different periods. For example, Grossman and Krueger analyzed on the relevance of environmental quality and economic development, finding that the relationship between them is an inverted U-shaped curve [49]. Per capita GDP is selected for eliminating the influence of population size [50]. In addition, the square of per capita GDP is introduced for exploring the relationship between the EWP and economic development level.

#### 3.2.2. Urbanization Level

Urbanization level affects the EWP through multiple mechanisms. The main view is that urbanization level promotes urban economic development and improves the energy and resource utilization efficiency, which has a positive effect on the EWP. However, some scholars argued that the low quality of urbanization causes large energy and resource consumption, and increases the pressures of urban environment, which has a negative effect on the EWP [51]. The proportion of urban population to total urban population at city level is selected to measure urbanization level. 

#### 3.2.3. Urban Compactness

Moderate urban scale can optimize the comprehensive benefits of a city. It is believed that urban compactness not only means providing more urban space with the least land, but also reflects the pursuit of a better quality of life in the city. In contrast, any scholars argued that strict planning of urban boundaries may be ineffective, and the establishment of the compact city is not an effective policy [52]. Referring the research of Long et al. [53], the population density is chosen to measure urban compactness in this paper.

#### 3.2.4. Industrial Structure

Industrialization has facilitated economic growth. However, it has aggravated environmental pollution due to high pollution and consumption, affecting people’s quality of life and health, and hindering the improvement of regional well-being. For example, manufacturing, power, gas and construction cause serious environmental pollution. Inspired by Zhong et al. [54], the proportion of added value of secondary industry to GDP at city level is used for measuring the industrial structure.

#### 3.2.5. Economic Extroversion

The “pollution paradise hypothesis” assumes that developed countries prefer to choose countries with relatively loose environmental regulation requirements to set up pollution-intensive enterprises [55]. In contrast, there is another hypothesis that economic extroversion prompts multinational enterprises to bring original capital, strengthen cooperation and connection of international enterprises through the spillover effect and competition effect. The economic extroversion is estimated by the proportion of foreign direct investment to GDP at city level in this paper.

#### 3.2.6. Urban Greening Level

There are many literatures have studied on urban greening. For example, Jin, et al. [56] explored the relevance between well-being and greening levels in South Korea, finding that greening level has a positive correlation on the well-being. Conversely, some scholars have analyzed the impact of urban greening on air quality, finding that the impact of the greening coverage rate on urban air is not significant [57]. Referring to the experience of Luo, et al. [58], the urban green area is selected for measuring the urban greening level.

#### 3.2.7. Technological Progress

The introduction of advanced technology, the innovation of management levels and improvement of labor quality can generate technology and knowledge spillover, and reduce the emission of environmental pollutants. However, some enterprises with low level of environmental pollution control and relatively low production capacity are not sensitive to technology input, and the cost of technology input forms a “crowding out effect” on enterprise production [59]. The technological progress is defined by the proportion of science and technology investment to GDP at city level in this paper. The driving factors of the EWP are shown as in Table 2.

## 4. Results and Discussion

### 4.1. Spatial and Temporal Evolution Characteristics on the EWP

#### 4.1.1. Analysis of the EWP Evaluation

This paper used MaxDEA 8 Ultra software to measure the EWP. The average EWP value was 0.6229, which was relatively low. Among 285 cities, Sanya, Shenzhen and Haikou ranked in the top three in terms of the EWP, and the average values were 1.870, 1.257 and 1.228, respectively. However, the EWP values of Guigang, Yichun and Baishan were 0.126, 0.124 and 0.106, respectively. This may be related to these top EWP performers benefiting from the rapid development of regional economy and the advance of technology.

**Figure 4:**
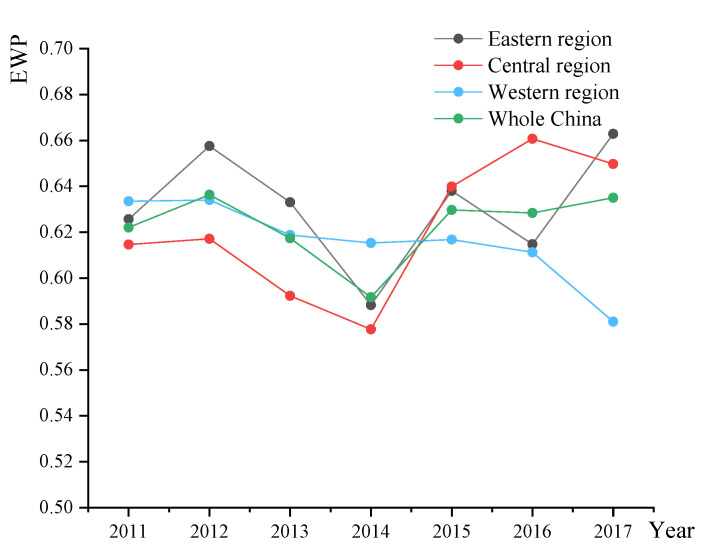
The EWP in Chinese eastern, central, western region and whole China from 2011–2017.

From Figure 4, it can be seen that the EWP fluctuates every year in China. The average EWPs of eastern, central and western cities were 0.6297, 0.6217 and 0.6158. From the perspective of regional differences, the EWPs show the decreasing pattern of “east-middle-west”. Relying on the physical and geographical advantages of coastal areas, the eastern region cities assimilate foreign investment. Most of them are exposed to high-end technology, forming a cumulative effect of economic, educational, medical and other advantages [66]. In contrast, the EWP of western cities is lower than that of other areas. A lot of cities in the western region are geographically faraway, and the transportation and education systems are underdeveloped, so this type of urban construction needs to promote high-quality economic development.

**Figure 5:**
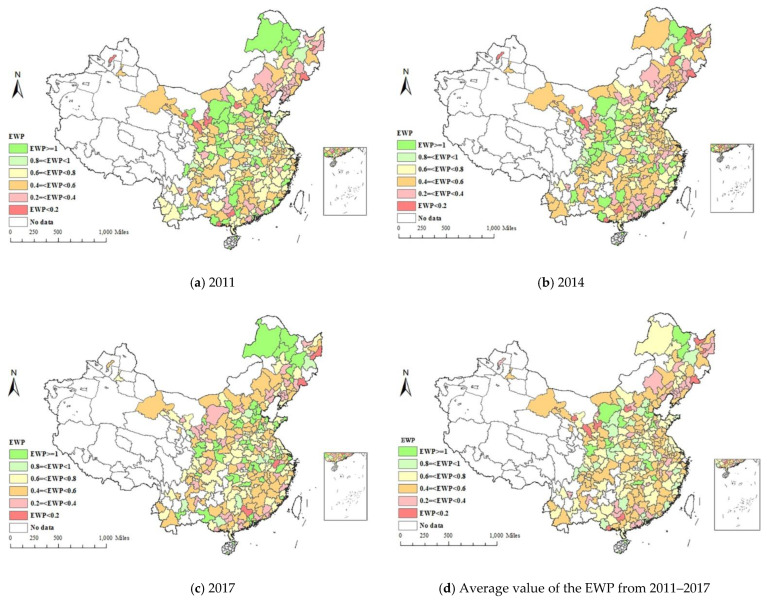
Spatial pattern of the urban EWP in 2011, 2014, 2017 and 2011–2017.

In this study, Arcgis software was applied to study the EWP space distribution of 285 prefecture-level cities in China. Figure 5a–d shows the spatial distribution of urban EWP in 2011, 2014, 2017 and 2011-2017. Figure 5a presents the cities such as Shenzhen, Guangzhou and other eastern cities, mainly distributed throughout the excellent group (EWP ≥ 1) in 2011. Compared with 2011, the number of cities of eastern region at the excellent group decreased in 2014. The number of central and western cities in the worst group (EWP < 0.2) decreased in 2017, indicating that China has made remarkable achievements in new urbanization development since 2014. It can be seen from Figure 5d that from 2011 to 2017, the average EWP values of central and western cities retain at relatively low level. The economic level of these cities is relatively dropped behind, and the limited environmental governance investment, the low levels of education and medical treatment are also the inducing factors. As a result of the unreasonable industrial structure and the relatively backward technical level, the ecological input and well-being output are not proportional.

#### 4.1.2. Spatial Autocorrelation Analysis

Spatial autocorrelation analysis was applied to explore the spatial distribution of the EWP in 285 Chinese cities from 2011 to 2017. Geoda software was adopted to analyze Moran’s I index of the EWP. The whole of Moran’s I values were greater than 0. Table 3 shows that the autocorrelation tendency of the EWP from 2011 to 2017 was roughly “up-down-up”.

(1) In 2011–2012, the Moran’s I value raised from 0.0869 to 0.1419, and was significant at a 1% level. The Moran’s I value was 0.1419, which implied that the positive spatial correlation of the EWP was very significant in 2012.
(2) The spatial correlation of the EWP has experienced a continuous decline from 2013–2016. The agglomeration degree of the EWP began to increase in 2017, indicating that the implementation achievements of new urbanization and relative policies have been gradually effective.

**Figure 6:**
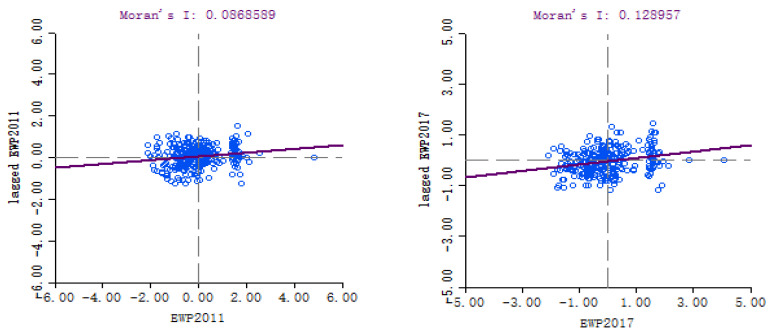
Moran scatter plots of the EWP in 2011 (**left**) and 2017 (**right**).

Since there existed spatial agglomeration of the EWP in Chinese prefecture cities, this paper further conducted Moran scatter plots from 2011 and 2017 to analyze the local spatial correlation of the EWP. Figure 6 shows that 151 cities (52.98%) have positive spatial dependence. Among them, 80 cities (28.07%) were located in quadrant I (HH type), 71 cities (24.91%) were located in quadrant III (LL type). The spatial correlation of the remaining 134 cities was not similar, and they accounted for 47.02%. The results show that most cities and their adjacent cities in China have similar spatial cluster characteristics to the EWP. Compared with 2011, the spatial agglomeration distribution characteristics of the EWP in 2014 were more significant. It shows that the green development achievements have been gradually obvious. The results show that 168 cities (58.95% of total number) have positive spatial correlation on the EWP. Among them, 68 cities (23.86%) and 100 cities (35.09%) were HH type and LL type in 2017, and 119 cities (41.05%) were LH type and HL type. It can be seen that Moran scatter plots have the following characteristics:

(1) In 2011, 2014 and 2017, the agglomeration types of the EWP in China was mainly HH type and L-L type. This indicated that the spatial dependence characteristics of the EWP was significant, and most cities and their neighboring cities show similar agglomeration characteristics.
(2) During the study period, HH type mainly concentrates in the eastern region. These cities have advantages in geographical location and resources. The HH types have high spatial agglomeration, and become the growth pole of driving the EWP in surrounding cities.
(3) LL type mainly appears in China’s heavy industry, energy base and western remote regions. Some cities in LL types have backward economy and fragile ecological environment, leading to the low value agglomeration of the urban EWP.

The Moran’s I index analysis implies that the clustering phenomenon of the urban EWP is significant. The traditional regression model usually ignores the spatial correlation and spatial heterogeneity, and the estimation results are not accurate enough. Therefore, the spatial econometric method was conducted to further test the spatial effects of driving factors on the EWP.

### 4.2. Analysis of Driving Factors on the EWP 

#### 4.2.1. Selection and Estimation of Spatial Panel Model

First, whether the model has spatial correlation was tested, and the OLS panel model with non-spatial effects was estimated (Table 4). From the result analysis of ordinary panel regression, LM Test and robust LM lag test rejected the original hypothesis of no spatial lag and no spatial error, and both of them were significant at 1% level. Consequently, only using the common OLS regression may cause the imprecise results. The spatial effect panel model was used to make spatial regression analysis, which is expressed as follows:

(9) $$EWP_{it}=\alpha +\beta (PGDP_{it},PGS_{it},UR_{it},\ln PD_{it},IS_{it},FDI_{it},UG_{it},TP_{it})\;+W_{ij}\theta (PGDP_{it},PGS_{it},UR_{it},\ln PD_{it},IS_{it},FDI_{it},UG_{it},TP_{it})+\rho W_{ij}EWP_{it}+c_{i}+\mu_{i}+\varepsilon_{it}$$

where, $EWP_{it}$ represents the EWP of the i city of the t time, and β is the regression parameters of the driving factors; $W_{i,j}$ is the adjacent space weight matrix, and the first-order spatial weight matrix of rook is selected in this study; ρ is the interaction parameter of the EWP in adjacent cities, θ is the influence parameter of driving factors of adjacent cities on the EWP, and $c_{i}$, $\mu_{t}$ are spatial and temporal effects respectively, which are random disturbance terms.

The results of SDM, SAR and SEM spatial panel models are as shown in Table 5. LR test and Wald tests were adopted to determine if the SDM could be simplified as SAR or SEM. The Wald test and LR test both rejected the original hypothesis. This implies that the SDM could not be simplified as a SEM and SAR. Hausman test statistics rejected the original hypothesis at 1% significance level test, indicating that the fixed effects should be chosen. The results reveal that the SDM model is more appropriate than SAR and SEM. Therefore, the SDM with double-fixed effects is considered in this paper.

(1) The coefficient of economic development level had a positive impact on the EWP. It implies that economic development still takes a significant part in improving the EWP, and urban economic development is the premise and basis to improve the EWP. Economic development can bring human social benefits and increase the well-being of urban residents. 

The coefficient of the square of per capita GDP was positive significantly at a 1% level. There was the U-shaped line between the economic development level and the EWP, proving that the EWP decreases with the increase of economic activity at the initial stage of development, and then the EWP gradually improves with the improvement of economic development.

(2) The coefficient of the urbanization level was −0.0041, and had significantly negative impacts on the EWP. On one hand, it is mainly attributed to the urban crowding caused by rapid transfer of population from rural areas to cities. Moreover, the extensive urbanization not only promotes the economic development, but also causes great pressure on the ecological resources and natural environment. The mismatches between the rapid expansion of urbanization and urban infrastructure, education and medical resources reduce the well-being of residents.

(3) The influence coefficient of population density was 0.046, and it was not significant. It demonstrated that the population density had a positive effect on the EWP, and this observation reflected that of Song, et al. [67]. The compact city can make efficient use of land resources and can provide urban residents with higher quality public infrastructure and municipal services.

(4) The industrial structure had a negative impact on the EWP, and it was significantly at 5% level, which was consistent with the literature of Fan et al. [68]. China’s urbanization development is mainly based on industrialization, and the industrial structure is detrimental to the EWP optimization. It is crucial to expedite the transformation and upgrade of industrial structure to improve the EWP.

(5) The coefficient of economic extroversion was significant at 10% level. It shows that China’s economic extroversion has an inhibitory effect on the urban EWP, indicating that China’s policies for introducing and utilizing foreign capital are relatively loose. Under the loose policy conditions, enterprises discharge a large number of pollutants, resulting in the reduction of the EWP.

(6) The coefficient of urban greening level was not significant. It indicated that the degree of urban greening had not taken a positive role in ameliorating the EWP, which is inconsistent with expectations. Generally speaking, the higher the urban greening level is, the higher the air quality might be. However, the regression result of urban greening level is not significant, which indicates that urban greening level is not balanced. For example, the amount of greenery in many urban centers and old districts is insufficient, and the overall urban greening level in the western region is lagging behind.

(7) A significant positive-correlation influence was found between the technological progress level and the EWP at 1% level. It implies that promoting the technological progress level is conducive to promote the EWP. China has increased the input of innovation resources, and the ability of independent innovation has been significantly enhanced in the late years. The high level of technological progress produces agglomeration and spillover effects, and improves the urban EWP.

#### 4.2.2. Analysis of Spatial Spillover Effect

The Matlab 2019a software was used to obtain the spillover effect of each variable (Table 6). The direct and indirect effects of per capita GDP were significant at 1% level. This implied that economic development had a positive spillover impact on the local EWP and a negative spillover effect on its adjacent cities. The total effect of per capita GDP was negative correlation and did not pass the significance level test. To some extent, it reflects that the city with higher economic development level may not have a positive effect on the EWP of adjacent cities.

The direct effect and total effect of urbanization development were significant at 1% level. It indicates that the low-quality urbanization development level has a negative impact on the local EWP and its adjacent cities. The low-quality urbanization mainly contains excessive of natural consumption and over-expanded urban construction. Urbanization development is a vital factor of the urban EWP.

The direct effect of industrial structure was significant at 5% level, indicating that the industrial structure had a negative effect on the local EWP. Although the increase of the secondary industry promotes the development of a local economy, it also brings environmental pollution, ecological deterioration and other environmental problems, which reduces the resource utilization efficiency and social well-being level. The coefficients of indirect effect and total effect were not significant.

The direct effect of economic extroversion was significant at 10% level, which implied that economic extroversion had a negative spillover effect on the local EWP. The indirect effect and total effect were not significant. It shows that foreign direct investment has caused environmental pollution, but the new technology and management experience have formed a diffusion effect in the adjacent cities.

The direct effect of technological progress was not significant. Its indirect effect coefficient had negative relation to the EWP. It shows that although technological progress promotes the EWP of the local region, it does not promote the EWP of surrounding cities. It has not played a positive role in driving the adjacent cities in the study period. This may be related to the adaptability of independent research and development achievements of some cities in China. The total effect of urban greening level was not significant. The direct effect and indirect spatial spatial of urban compactness on the EWP were not significant, and the multifaceted role of urban compactness was not fully reflected. 

#### 4.2.3. Analysis of Robustness

The robustness of SDM is tested by changing spatial weight, and the adjacent space–weight matrix is replaced by the distance–space–weight matrix. The coefficient significances of driving factors on the EWP are basically consistent with Table 7, which further confirms that the results of this study are basically credible. The analysis results of robustness test prove that after changing the spatial weight, the EWP regression result of SDM with the adjacent space weight matrix is robust.

## 5. Conclusions and Policy Implications

### 5.1. Conclusions

The spatial and temporal evolution characteristics of 285 Chinese prefecture level cities were analyzed from 2011–2017. The SDM was built to reveal the driving factors of the EWP with the spatial panel data. The main conclusions showed that: 

First, from the evaluation of the EWP, the average EWP value of 285 Chinese cities was 0.6229, and the efficiency value was relatively low. The trend of overall EWP has experienced a stage evolution process of “upward → downward → upward”. The cities of average EWP in eastern and central region rated first and second, and cities in western region ranked third according to the EWPs. Due to the coastal geographical advantages and priority support of policies, the levels of economy, education and medical care in the eastern region are evidently superior. 

Second, from the spatial autocorrelation analysis, it is reasonable to conclude that Moran’s I index of the EWP during the study period was greater than 0. There was spatial agglomeration of the EWP in 285 Chinese cities, and the spatial effect was significantly positive. The spatial agglomeration types of the EWP were mainly HH type and LL type. The urban EWPs have significant spatial agglomeration and path dependence. 

Third, the economic development level and technological progress had significant roles in promoting the EWP. The urbanization level, industrial structure and economic extroversion had negative effects on the EWP. The impacts of urban compactness and urban greening level on the EWP were not significant. The negative spatial spillover effect of urbanization level on the EWP was significant.

Nonetheless this study has some deficiencies, and the topic needs further research. With regard to aspects of the EWP evaluation, there are some limitations in data collection of city scale. When these data can be developed in the future, the evaluation indicators need to be enriched, such as the education development level, medical development level and subjective well-being indicators. The time span of the EWP established in this paper only includes 7 years. If the follow-up research can establish the EWP time series with a time span of 10–20 years, it can further study the spatial and temporal evolution pattern of the EWP scientifically. The driving factors of the EWP only analyze social and economic factors such as economic development level, urbanization level and so on, but not include natural environmental factors such as climate and altitude.

### 5.2. Policy Implications

First, it is found that there are positive spatial autocorrelation and path dependence of the EWP. The cities in the eastern region have higher economic, educational and medical level, and superior environmental supervision. The EWPs of 285 Chinese prefecture-level cities have obvious spatial correlations. Urban agglomerations in the eastern region should continue to maintain the spatial agglomeration mode of high EWP value, and promote the coupling development of ecology and economy. The regions with low EWP should avoid the diffusion of LL type clusters and improve the ability of independent innovation.

Second, the range of ecological environment and resource-carrying capacity should be promoted. It is therefore recommended that the government solve the problem of agricultural transfer population, improve medical and educational conditions, and enhance the construction of urban infrastructure.

Finally, the technological innovation of ecological environment should be improved. The incentive policies for technological innovation need to be improved, such as tax incentives, innovation subsidies and so on. In addition, the transformation achievements of science and technology in the ecological environment should be strengthened. The scientific and technological support systems for ecological environments need to be built.

## Figures and Tables

**Table 1:** The indicator descriptions of the EWP evaluation.

Dimension	Criteria	Indicators	Unit
Input indicators	Ecological input	Per capita urban electricity consumption ($x_{1}$)	Kwh
		Per capita water consumption of residential use ($x_{2}$)	Ton
		Per capita built-up area ($x_{3}$)	${\;m}^{2}$
Output indicators	Well-being	Per capita GDP ($y_{1}^{d}$)	Yuan
		The number of students enrolled per $10^{4}$ persons ($y_{2}^{d}$)	Person
		The number of doctors per $10^{4}$ persons ($y_{3}^{d}$)	Year
	Environment pollutants	Per capita wastewater discharge ($y_{1}^{\mathrm{ud}}$)	Ton
		Per capita SO_2_ ($y_{2}^{\mathrm{ud}}$)	kg
		Per capita soot /dust ($y_{3}^{\mathrm{ud}}$)	kg

Note: In order to negate the influence of inflation, the economic data of the GDP were normalized to that of 2011.

**Table 2:** The definition of variables.

Explanatory Variables	Definitions of Variables	Variable Abbreviation	Key References
Economic development level	Per capita GDP (10^4^ yuan/person)	PGDP	[60]
	(Per capita GDP)^2^	PGS	
Urbanization level	The proportion of urban population to total urban population (%)	UR	[61,62]
Urban compactness	The population density(Person/square kilometer)	PD	[53]
Industrial structure	The proportion of added value of secondary industry to GDP (%)	IS	[54]
Economic extroversion	The proportion of foreign direct investment on GDP (%)	FDI	[63]
Urban greening level	Per capita urban green area (m^2^/person)	UG	[64]
Technological progress	The proportion of science and technology investment on GDP (%)	TP	[65]

**Table 3:** Moran’s I statistical values for the EWP of 285 Chinese cities from 2011 to 2017.

Year	Moran’s I	*p* Value	Z Value
2011	0.0869	0.0070	2.3557
2012	0.1419	0.0010	3.5734
2013	0.1032	0.0030	2.7583
2014	0.0885	0.0100	2.3945
2015	0.0210	0.2530	0.6357
2016	0.0178	0.2840	0.6055
2017	0.1290	0.0010	3.4757
2011–2017	0.0900	0.0060	2.4520

**Table 4:** The non-spatial panel model test results of the EWP.

Statistic	Mixed Effect	Spatial Fixed	Time Fixed	Double-Fixed
Adjusted R^2^	0.1740	0.0508	0.1830	0.0576
$\sigma^{2}$	0.0595	0.0172	0.0586	0.0168
loglikfe	−11.4752	24.7052	2.5676	25.1069
LM Lag	71.1922 ***	53.3217 ***	72.0546 ***	40.2838 ***
Robust LM Lag	39.6311 ***	11.1342 ***	31.5794 ***	6.5575 ***
LM Error	131.0704 ***	65.6392 ***	126.7096 ***	49.2843 ***
Robust LM Error	99.5093 ***	23.4518 ***	86.2344 ***	15.5580 ***

Note: *** indicates significant level of 1%.

**Table 5:** The spatial effect panel regression results of the EWP.

Variables	SDM		SAR		SEM	
	Coefficients	T	Coefficients	T	Coefficients	T
PGDP	0.0174 ***	3.6777	0.0084 **	2.1429	0.0106 **	2.5289
PGS	0.0222 ***	3.6157	0.0249 ***	4.6640	0.0254 ***	4.4866
UR	−0.0041 ***	−4.9031	−0.0043 ***	−5.0739	−0.0042 ***	−4.9428
ln PD	0.0460	0.7145	0.0231	0.3682	0.0343	0.5376
IS	−0.0021 **	−2.0816	−0.0021 **	−2.3939	−0.0022 **	−2.3868
FDI	−0.0028 *	−1.9392	−0.0026 *	−1.8408	−0.0027 *	−1.9149
UG	−0.0005	−2.0442	−0.0005	−2.0198	−0.0006	−2.2200
TP	0.0597 ***	4.9796	0.0564 ***	4.8698	0.0585 ***	4.94709
W× PGDP	−0.0257 ***	−3.110				
W× PGS	−0.0029	−0.2805				
W× UR	−0.0015	−0.8384				
W× ln PD	−0.2228	−1.4155				
W× IS	0.0020	1.1574				
W× FDI	0.0032	1.2116				
W× UG	0.0011 **	2.0626				
W× TP	0.0290	1.3504				
$\sigma^{2}$	0.0188		0.0191		0.0190	
R-squared	0.7764		0.7724		0.7660	
Log-likehood	1282.9770		1265.5207		1269.5204	
**Statistical Tests**	**Z-Value**	***p*-Value**				
Wald_spatial_lag	30.2098 **	0.0355				
LR_spatial_lag	34.9126 **	0.0187				
Wald_spatial_error	22.9044 ***	0.0035				
LR_spatial_error	26.9133 **	0.0134				
Hausman test	−68.0646 ***	0.0000				

Note: * indicates significant level of 10%; ** indicates significant level of 5%; *** indicates significant level of 1%.

**Table 6:** The spatial spillover effect of the EWP.

Variables	Direct Effect		Indirect Effect		Total Effect	
	Coefficient	T	Coefficient	T	Coefficient	T
PGDP	0.0167 ***	3.6381	−0.0274 ***	−2.8400	−0.0108	−1.2248
PGS	0.0220 ***	3.7379	0.0021	0.1711	0.0241 *	1.9455
UR	−0.0042 ***	−5.0343	−0.0030	−1.3533	−0.0072 ***	−2.9001
ln PD	0.0381	0.6219	−0.2616	−1.3815	−0.2235	−1.1584
IS	−0.0020 **	−2.0481	0.0020	0.9272	−0.00004	−0.0197
FDI	−0.0030 *	−1.8749	0.0032	0.9610	0.0006	0.1698
UG	−0.0005 *	−1.9544	0.0012 *	1.8744	0.0007	0.9750
TP	0.0203	−5.0581	−0.0595 *	0.7982	−0.0391	−1.4223

Note: * indicates significant level of 10%; ** indicates significant level of 5%; *** indicates significant level of 1%.

**Table 7:** The analysis results of robustness test.

Variables	SDM		SLM		SEM	
	Coefficients	T	Coefficients	T	Coefficients	T
PGDP	0.0164 ***	3.3192	0.0074 *	1.8925	0.0095 **	2.2372
PGS	0.0239 ***	3.9582	0.0257 ***	4.7986	0.0263 ***	4.6925
UR	−0.0041 ***	−4.8689	−0.0043 ***	−5.1489	−0.004 ***	−5.0713
ln PD	0.0380	0.5962	0.0298	0.4722	0.0372	0.5850
IS	−0.0015 **	−1.5868	−0.0020 *	−2.2145	−0.0019 **	−2.1001
FDI	−0.0026 *	−1.7957	−0.0026 *	−1.8719	−0.0027 *	−1.8616
UG	−0.0004	−1.6258	−0.0005	−2.0074	−0.0005	−2.0798
TP	0.0577 ***	4.9013	0.0565 ***	4.8537	0.0585 ***	4.9841
W× PGDP	−0.0164 ***	−2.7416				
W× PGS	−0.0030	−0.3334				
W× UR	−0.0009	−0.5931				
W× ln PD	−0.1382	−1.1232				
W× IS	0.0004	−0.2447				
W× FDI	0.0024	0.7403				
W× UG	0.0008 **	1.9851				
W× TP	0.0276	1.5969				
$\sigma^{2}$	0.0190		0.0193		0.0192	
R-squared	0.7738		0.7706		0.7660	
Log-likehood	1274.9153		1260.6148		1263.5959	
**Statistical Tests**	**Z-Value**	***p*-Value**				
Wald_spatial_lag	24.6256 ***	0.0018				
LR_spatial_lag	28.6011 ***	0.0004				
Wald_spatial_error	19.7626 **	0.0113				
LR_spatial_lag	22.6389 ***	0.0039				
Hausman_ test	−35.2411 ***	0.0058				

Note: * indicates significant level of 10%; ** indicates significant level of 5%; *** indicates significant level of 1%.

## Data Availability

Not applicable.
